# Malaria epidemiological characteristics and control in Guangzhou, China, 1950–2022

**DOI:** 10.1186/s12936-023-04696-y

**Published:** 2023-09-11

**Authors:** Yuehua Chen, Hao Zhang, Haiyan Chen, Lirui Fan, Conghui Xu, Jianmin Xu, Shouyi Chen, Kuncai Chen, Yuehong Wei

**Affiliations:** 1 Institute of Public Health, Guangzhou Medical University, Guangzhou, China; 2 Department of Parasite and Endemic Disease Prevention and Control, Guangzhou Center for Disease Control and Prevention, Guangzhou, China

**Keywords:** Malaria, Epidemiology, Control strategies, Vector control, Elimination

## Abstract

**Background:**

Malaria was once widespread in Guangzhou, China. However, a series of control measures have succeeded in eliminating local malaria infections. Based on the analysis of the characteristics of malaria epidemics in Guangzhou, China, from 1950 to 2022, the changes and effectiveness of malaria control strategies and surveillance management in Guangzhou from 1950 to 2022 are described.

**Methods:**

Data on malaria prevention and treatment in Guangzhou from 1950 to 2022 were collected, and descriptive epidemiological methods were used to analyse the prevalence of malaria, preventive and control measures taken, and the effectiveness of prevention and treatment in different periods. Data on malaria cases were obtained from the Guangzhou Centre for Disease Control and Prevention (CDC) and the China Communicable Disease Reporting System.

**Results:**

The development of the malaria control system in Guangzhou has gone through four periods: 1. High malaria prevalence (1950–1979), 2. Intensive prevention and control stage (1980–2000), 3. Consolidating gains in malaria control (2001–2008), and 4. Preventing reestablishment of transmission (2009–2022). During Period 1, only medical institutions at all levels and the local CDCs, the Guangzhou CDC participated in the malaria prevention and control system, establishing a three-tier health system on malaria prevention and control. During Period 2, other types of organizations, including the agricultural sector, schools and village committees, the construction department and street committee, are involved in the malaria control system. During Period 3, more and more organizations are joining forces to prevent and control malaria. A well-established multisectoral malaria control mechanism and an improved post-elimination surveillance management system are in place. Between 1950 and 2022, a total of 420,670 cases of malaria were reported. During Period 1, there was an epidemic of malaria in the early 1950s, with an annual incidence rate of more than 10,000/100,000, including a high rate of 2887.98/100,000 in 1954. In Period 2 malaria was gradually brought under control, with the average annual malaria incidence rate dropping to 3.14/100,000. During Period 3, the incidence rate was kept below 1/100,000, and by 2009 local malaria infections were eliminated.

**Conclusion:**

For decades, Guangzhou has adopted different malaria control strategies and measures at different epidemic stages. Increased collaboration among civil organizations in Guangzhou in malaria control has led to a significant decline in the number of malaria cases and the elimination of indigenous malaria infections by 2009.The experience of Guangzhou can guide the development of malaria control strategies in other cities experiencing similar malaria epidemics.

## Background

Malaria is a parasitic disease caused by a complex of pathogens of the genus *Plasmodium*, with *Anopheles* mosquitoes as the vector of transmission [1–4]. Malaria causes intermittent fever and a range of clinical syndromes that, if left untreated, can rapidly progress to serious illness, most notably the devastating neurological complications caused by cerebral malaria [5]. According to the World Health Organization (WHO), the number of malaria cases in 85 malaria-endemic countries was estimated at 241 million globally in 2020, up from 227 million in 2019, with Africa accounting for approximately 95% of the total number of malaria cases [6]. Most deaths occur in children under 5 years of age and are mainly caused by *Plasmodium falciparum* in sub-Saharan Africa [7–10]. Malaria has complex epidemiological factors and remains a major public health concern and one of the priority statutory infectious diseases for global control [11, 12].

In China, malaria has been endemic for at least 3000 years and has wreaked havoc on people’s health and productive activities [13]. It is estimated that before 1949, more than 350 million people out of a total population of about 450 million were threatened by malaria, with at least 30 million malaria cases per year and a mortality rate of about 1%. Following a major national effort to reduce the malaria burden, malaria transmission has been interrupted, with zero local cases reported since 2017 [14, 15]. Guangzhou used to be one of the more serious malaria epidemics, especially in rural areas and mountainous forests, where malaria is widely distributed and has a high incidence rate [16, 17]. After years of malaria prevention and control, with the improvement of the multisectoral mechanism and the monitoring and management system, the incidence of malaria has been declining year by year, and indigenous malaria infections have been eliminated by 2009 [18].

To accelerate progress toward malaria elimination and respond positively to the WHO strategic goal of reducing global malaria morbidity and mortality by at least 90% by 2030, some countries have explored different approaches to malaria elimination. There is growing awareness of an interdisciplinary approach to disease prevention and control, known as “One Health” [19], which is based on vector control and population and environmental prevention integrated with the diagnosis and treatment of malaria in health care facilities and local, national, and global leadership to implement and fund malaria control programmes [20–23]. This approach requires the collaborative efforts of public health professionals, doctors, and staff from multiple disciplines and relevant sectoral agencies. The One Health approach has been applied in several countries in Africa, South Asia, and Latin America [24].

The Chinese and local governments prioritized malaria prevention and control and have implemented several strategies to control malaria and prevent human infections [25]. The large-scale deployment of malaria prevention and treatment technologies, coupled with multi-sectoral, integrated prevention and treatment, has led to substantial expansion of malaria control work in Guangzhou, with improved results in all aspects of malaria elimination [26]. In 2021, the WHO awarded China national malaria elimination certification, and no cases of indigenous primary malaria infection have been detected in China for 4 consecutive years [6]. The purpose of this paper is to review the malaria control strategies and measures implemented in Guangzhou in recent decades, and to provide a reference for other cities that are facing challenges in developing effective malaria control strategies.

## Methods

### Study area

**Fig. 1:**
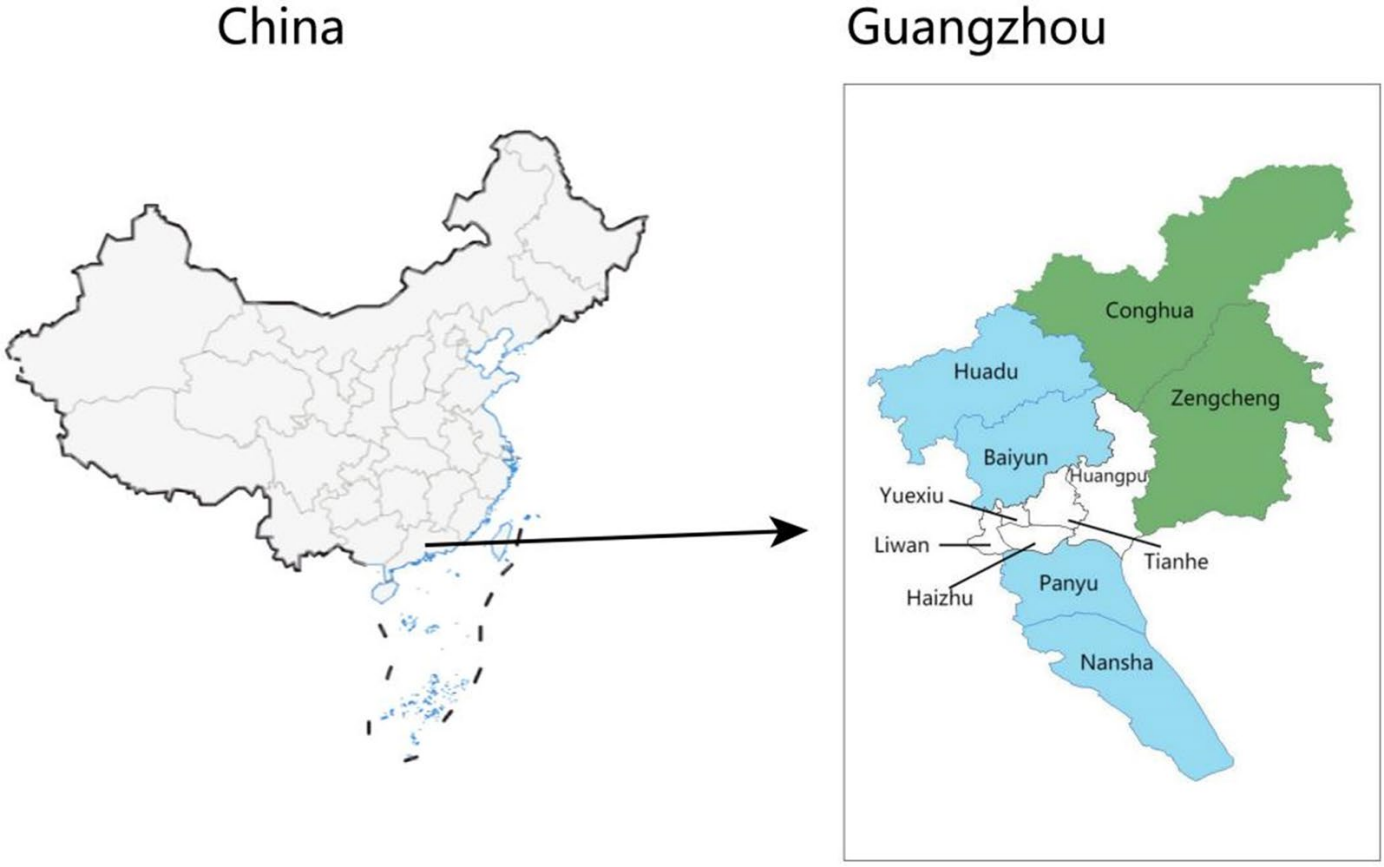
Administrative districts, Guangzhou, 2022. Guangzhou City consists of five urban areas (white), four rural areas (blue), and two satellite cities (green)

Guangzhou is the capital of Guangdong Province and is located in the south–central part of the province, in southern China (Fig. 1). Guangzhou is located on the subtropical coast, with the Tropic of Cancer passing through the south–central part of the city. Guangzhou has a maritime subtropical climate, characterized by warmth and rain, abundant light and heat, long summers, and short frost periods. The average temperature throughout the year is 21.5–22.2 ℃, the average relative humidity is 77%, there are approximately 150 days of precipitation annually, and average annual rainfall is approximately 1800 mm. The year-round rains and heat are abundant and suitable for the breeding of malaria and other disease vectors. The city covers an area of 7434.4 km^2^, including two satellite cities (Zengcheng and Conghua), five urban administrative regions (Huangpu, Tianhe, Yuexiu, Liwan, and Haizhu), and four rural administrative regions (Panyu, Huadu, Baiyun, and Nansha). Guangzhou City consists of five urban areas (white), four rural areas (blue), and two satellite cities (green).

**Fig. 2:**
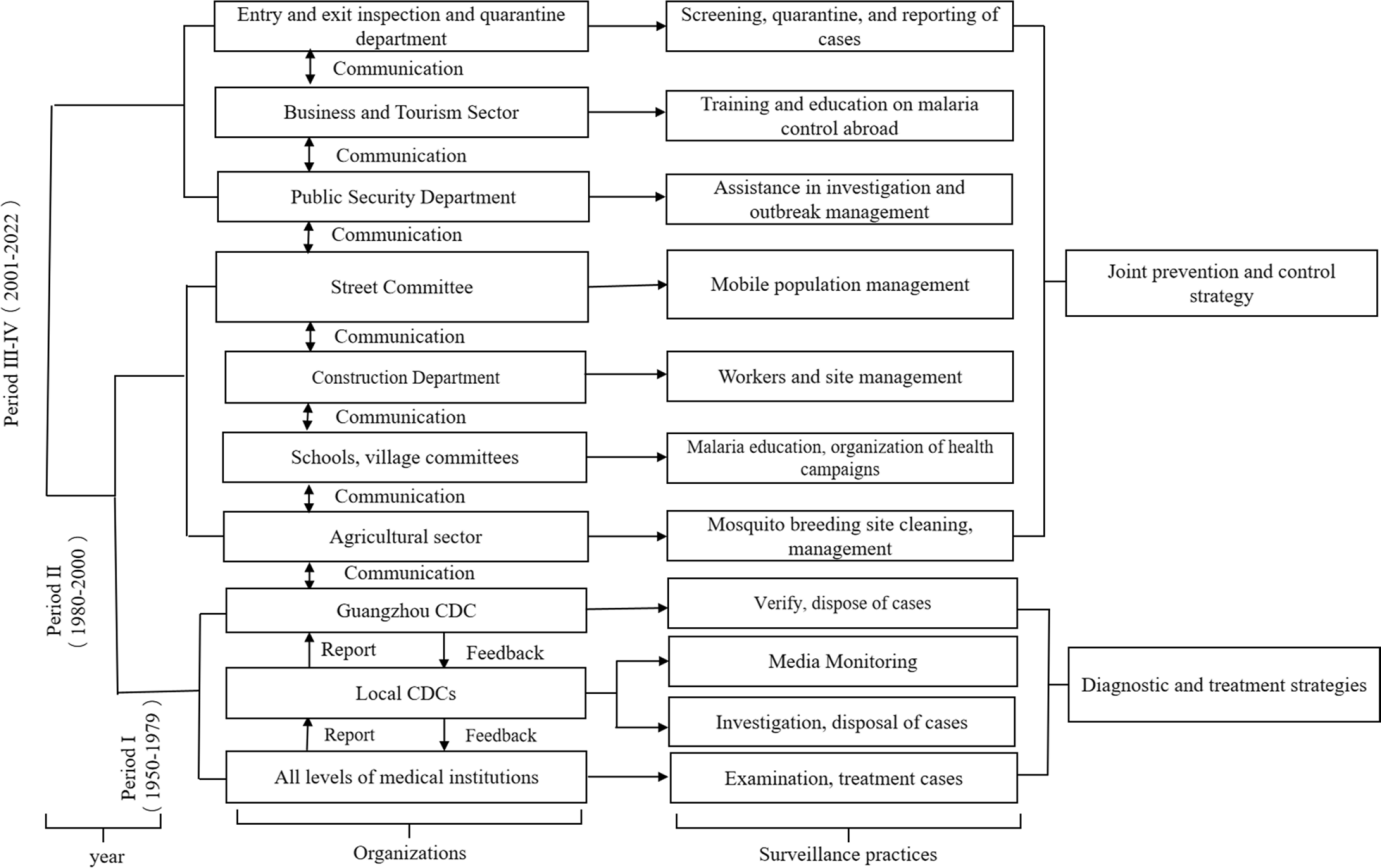
Development of the malaria control system in Guangzhou, China, 1950–2022

The current malaria surveillance and management system in Guangzhou has been continuously developed and improved over decades and comprises two main components: diagnosis and treatment by the medical and health sectors, and joint prevention and control management by various administrative departments (Fig. 2). Medical institutions at all levels are responsible for identifying and treating malaria cases and reporting them to local centres for disease prevention and control (CDC). Each local CDC conducts vector investigation and control, disposal, and reporting to the Guangzhou Centre for Disease Control and Prevention, which is responsible for malaria case verification, epidemiological investigation, outbreak site investigation, and management. Local agricultural departments are responsible for cleaning and managing mosquito breeding sites. Schools and village committees are responsible for malaria prevention and control education and organizing public health campaigns. The construction department is responsible for construction workers and site management. Street and neighborhood committees are responsible for mobile population management. The public safety department is responsible for assisting in the investigation and disposal of the epidemic. The commercial tourism sector is responsible for educating and training Chinese people traveling abroad regarding malaria prevention and control. The immigration inspection and quarantine department is responsible for screening, quarantining, and the reporting of cases. Long-term cooperation and communication among organizations is needed for joint malaria control and prevention.

### Case definition

The World Health Organization malaria reporting guidelines recommend malaria diagnosis through microscopy or rapid diagnostic tests for malaria and effective anti-malarial treatment, and blood smear microscopy is considered the gold standard for malaria diagnosis [27]. The definition of malaria in this study followed the malaria diagnostic criteria developed by the National Health and Family Planning Commission of the People’s Republic of China (WS259-2015) [28]. A clinically diagnosed case is defined as a person who has had a history of overnight stay in a malaria-endemic area or a history of blood transfusion within the last 2 weeks, and who presents with typical clinical symptoms, such as periodic chills, fever and sweating. A clinically diagnosed case is transited into a confirmed malaria case when one of the following tests is positive: (i) microscopic examination of blood smears to detect *Plasmodium*; (ii) a positive test for Plasmodium antigens; (iii) a positive test for *Plasmodium* nucleic acids.

### Data analysis

All malaria case data were obtained from the health statistics yearbook of the Guangzhou Municipal Centre for Disease Control and Prevention (formerly the Guangzhou Municipal Health Epidemiology Station), data compilations, statistical information on outbreaks, and the China Communicable Disease Reporting System (China Center for Disease Control and Prevention). Population statistics were obtained from the Guangzhou Statistical Yearbook (Guangzhou Municipal Bureau of Statistics). In the present study, the initial data were entered into the database using EpiData Entry v. 3.1 (EpiData, Odense, Denmark) and then analysed and described using R v. 3.31 (The R Foundation, Vienna, Austria). Descriptive statistics were used to summarize and analyse the epidemiological characteristics of malaria, prevention and treatment strategies and measures, and prevention and treatment effects in Guangzhou City in the years since 1950.

## Results

### Development of malaria control system

#### Period 1: high malaria prevalence (1950–1979)

The initial malaria case reporting system was established in 1950, collecting information on malaria cases from hospitals, health centers at all levels, traveling medical teams, and grassroots malaria blood testing stations. During Period 1, a three-tier health system for malaria prevention and treatment has been established, based on medical institutions at all levels, local centres for disease prevention and control and the Guangzhou Centre for Disease Prevention and Control (Fig. 2). Comprehensive malaria census, timely reporting and treatment of detected malaria cases, national prevention of medication, and radical treatment in resting stage. Health workers are trained at all levels to distribute medication, deliver it to homes, and spray insecticides or distribute insecticide-soaked mosquito nets. Carry out patriotic health campaigns to prevent and eliminate mosquitoes and cut off the means of transmission. Indoor residual spraying is carried out according to vector characteristics and *Plasmodium* species, and vector species and density testing is routinely performed. Strengthening health education on malaria prevention and treatment. Malaria control includes epidemiological investigation, post-exposure treatment of malaria, determination of the source of infection, vector control, investigation and management of outbreak sites, and health education.

#### Period 2: intensive prevention and control stage (1980–2000)

**Fig. 3:**
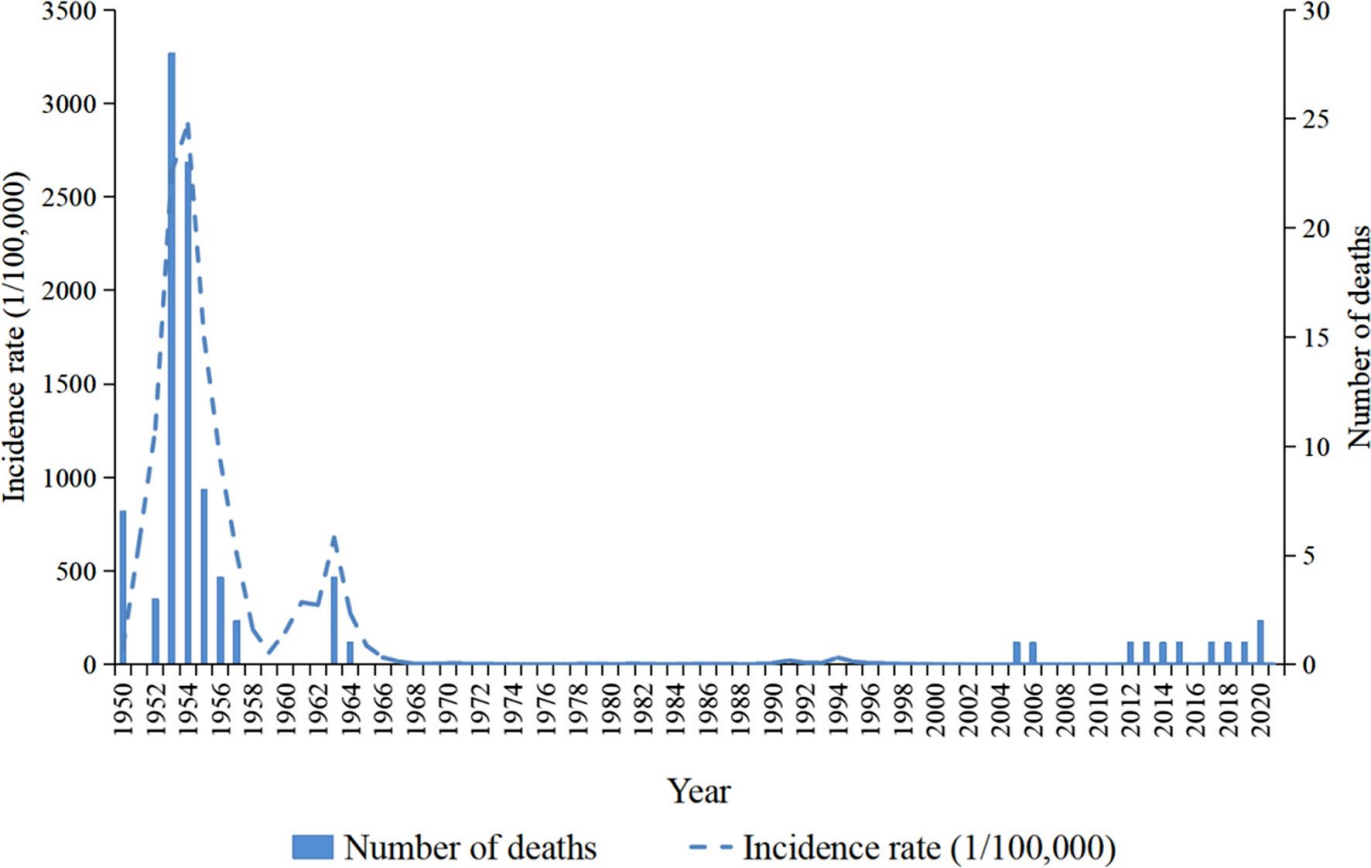
Trends in the incidence of malaria in Guangzhou, China, 1950–2021

With the entry of new sectors and the implementation of new measures, the number of malaria cases in the period 1980–2000 was significantly lower than in the previous period (Fig. 3). During 1980–2000, to improve knowledge among the general public regarding malaria prevention and treatment, health education activities on malaria were carried out by the Education Department, schools and village committees in each area, and public health campaigns on malaria were organized.

Malaria vector mosquitoes tend to breed in ponds and paddy fields [29]. In 1982, systematic surveillance was established in Guangzhou, including blood testing of febrile patients (1. history of malaria; 2. history of residence in a malaria-endemic area; 3. history of blood transfusion in the last 2 weeks; 4. fever of unknown cause), vector density and species determination. The agriculture department dredges ditches and cleans the city's water network to reduce mosquito breeding sites and habitats and reduce human–mosquito contact. Because many vector mosquitoes consume both human and animal blood, livestock fencing is built on the edge of villages, close to mosquito breeding sites. The use of livestock to attract mosquito vectors can serve as a protective barrier for the human population, reducing the likelihood of malaria mosquitoes biting humans. Indoor residual spraying (IRS) of livestock housing, such as cattle barns, is applied to lure and kill mosquitoes [30].

Strengthening malaria control in mobile populations is a priority for malaria control. In the late 1980s, a large number of migrant workers relocated to Guangzhou, leading to a rapid increase in malaria cases. Provisional measures for the management of malaria among the floating population were introduced in Guangzhou in 1985 [31]. Guangzhou has established a construction department to improve the management of construction workers and sites. This involves investigating and treating patients with a history of malaria and existing patients, formulating rules for the implementation of malaria prevention and treatment along railway lines, promoting the correct use of mosquito nets, preventing the importation of external sources of infection, and reducing the incidence of disease among mobile populations. All streets, neighbourhood committees, and other sectors are required to register mobile populations, and there is multisectoral cooperation to control malaria epidemics. By 1999, the incidence of malaria fell to 2.47/100,000, and malaria was basically under control. During this period, seven types of organization were involved in malaria management and surveillance: agricultural departments, school and village committees, building departments, street committees, medical institutions, district disease prevention and control centres, and the Guangzhou Centre for Disease Control and Prevention.

#### Period 3: consolidating gains in malaria control (2001–2008)

During this phase, the results of malaria control continue to be consolidated, with a comprehensive prevention and treatment strategy that focuses on controlling the source of infection. Comprehensive malaria case and mosquito vector surveillance. Actively carry out blood tests for febrile patients (1. clinically diagnosed malaria; 2. clinically suspected malaria; 3.fever of unknown cause) and conscientiously implement *Plasmodium* microscopy for febrile patients, so as to detect malaria pathogens in a timely manner and prevent the importation and spread of pathogens. The density, population structure and ecological environment of *Anopheles* mosquitoes are monitored, and the monitoring points are constantly adjusted according to the needs of urbanization and development. Strengthen the verification and reporting of malaria cases in various medical institutions. Since 2003, the first physician who detects a malaria case is required to implement web-based reporting through the Chinese CDC system within 24 h. Enhanced vector control through pharmaceutical indoor residual spraying in all outbreak sites based on vector characteristics and *Plasmodium* species. The malaria control system has reached further improvement. The last locally acquired case occurred in Guangzhou in 2008, and no locally acquired malaria case has been reported since then.

#### Period 4: preventing reestablishment of transmission (2009–2022)

Characterization of malaria cases in Guangzhou City over the period 2009–2022 as predominantly imported from outside the country. The main measures taken are to prevent the occurrence of locally infected cases caused by imported cases and to strengthen case surveillance, with a focus on improving the accuracy and timeliness of case surveillance, so as to achieve early detection, early treatment and early disposal. With the strengthening of the Malaria Surveillance Management System, the implementation of surveillance and response has been standardized into the “1-3-7” surveillance methodology for the identification of local and imported malaria cases [32]. Strengthen cooperation with the Entry-Exit Inspection and Quarantine Bureau (CIQ), the Ministry of Commerce, the Public Security Department and other departments to carry out joint prevention and control, and strictly implement the reporting of cases and investigation of individual cases. The CIQ provides training and education in malaria prevention and control for people going abroad and conducts malaria screening for people with fever returning from malaria-endemic areas abroad. Any positive malaria cases detected are promptly communicated to the local health planning committee, the Centre for Disease Control and Prevention, and medical institutions, which work closely in follow-up diagnosis, treatment, and long-term follow-up. The Ministry of Commerce has conducted training for migrant workers sent to malaria-endemic areas abroad to disseminate information regarding precautions for malaria prevention and control. The public security department assists in the investigation of malaria cases and infections among fellow travelers and in the emergency response to malaria outbreaks. Ongoing surveillance of febrile patients and blood tests to detect malaria pathogens in a timely manner. Continuous malaria vector surveillance. Strengthening training in malaria control skills and malaria microscopy skills to raise awareness of malaria diagnosis in health facilities. These measures have increased the sensitivity of the malaria surveillance system and achieved a rapid response to imported malaria cases. Multi-sectoral cooperation and communication have been strengthened and comprehensive malaria surveillance and management systems have been established. These organizations include: (1) entry–exit inspection and quarantine departments; (2) commercial tourism departments; (3) public security departments; (4) street and neighbourhood committees; (5) construction departments; (6) School and village councils; (7) agricultural departments; (8) medical institutions at all levels; (9) local disease prevention and control centres; and (10) the Guangzhou Centre for Disease Prevention. An extensive joint prevention and control mechanism has been established, with the timely transmission of information on epidemic developments, disease epidemic prevention and control, malaria importation in each district, timely follow-up treatment, enhanced vector surveillance, and environmental management to prevent the spread and prevalence of malaria.

### Human malaria cases

From 1950 to 2021, a total of 420,670 cases of locally acquired malaria were recorded in Guangzhou (Fig. 3). The highest incidence was 2887.98/100,000 in 1954, with 87,838 cases of malaria. There were three malaria outbreaks in Guangzhou during the twentieth century. The first peak in incidence was during the 1950s. The second peak was in the early 1960s, when the annual incidence rose from 318.28/100,000 in 1962 to 677.68/100,000 in 1963. The epidemic was brought under control in 1966, when the incidence dropped to 37.58/100,000. The third peak was in the early 1990s, with outbreaks occurring in 1991 and 1994, and incidence rates rising from 8.33/100,000 in 1990 to 21.67/100,000 in 1991 and from 8.31/100,000 to 37.02/100,000 in 1993. During Period 2, there was a clear declining trend of malaria incidence in Guangzhou during the 1980s, with a significant decrease compared with the previous period; the average annual incidence of malaria was maintained at 3.14/100,000. During Period 3, after 2000, the incidence rate remained below 1 per 100,000. Since 2009, there have been no local cases of malaria infection. During Period 4, all the malaria cases were imported cases. Malaria patient deaths were concentrated during the malaria outbreak epidemics of the mid-1950s and early 1960s.

**Fig. 4:**
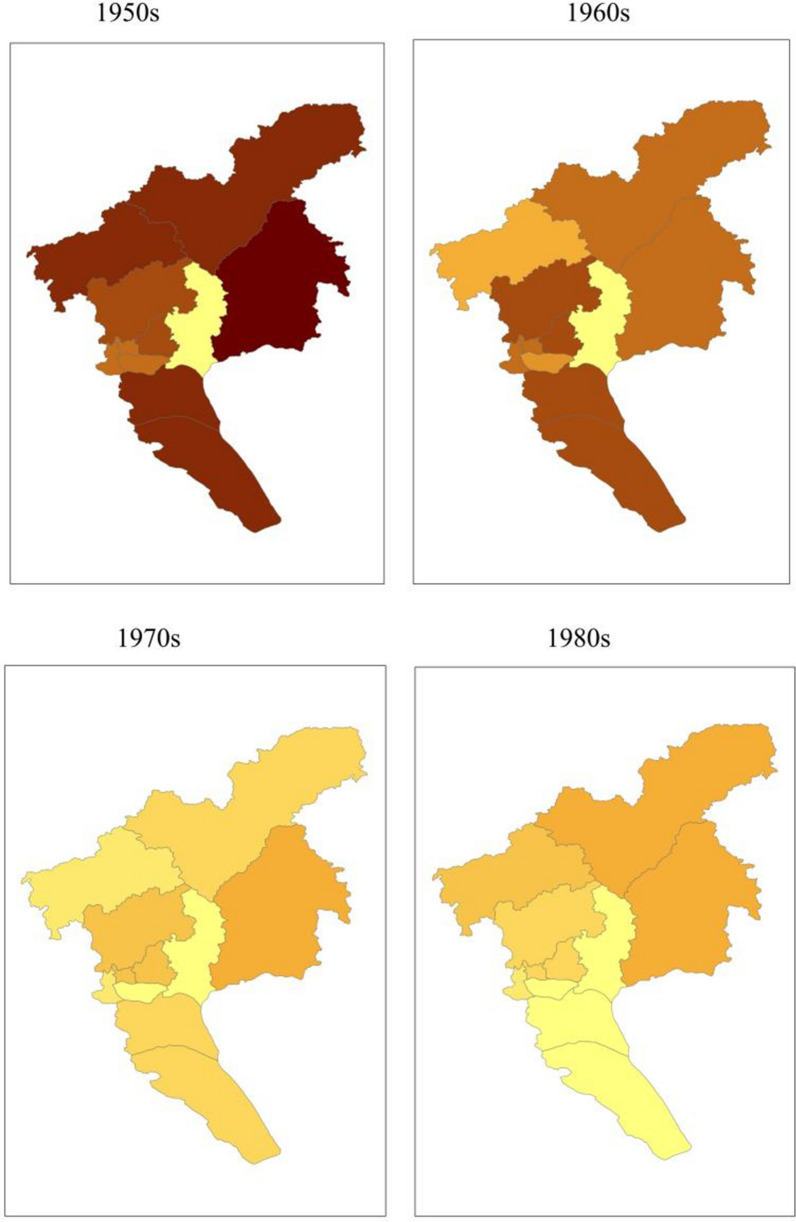

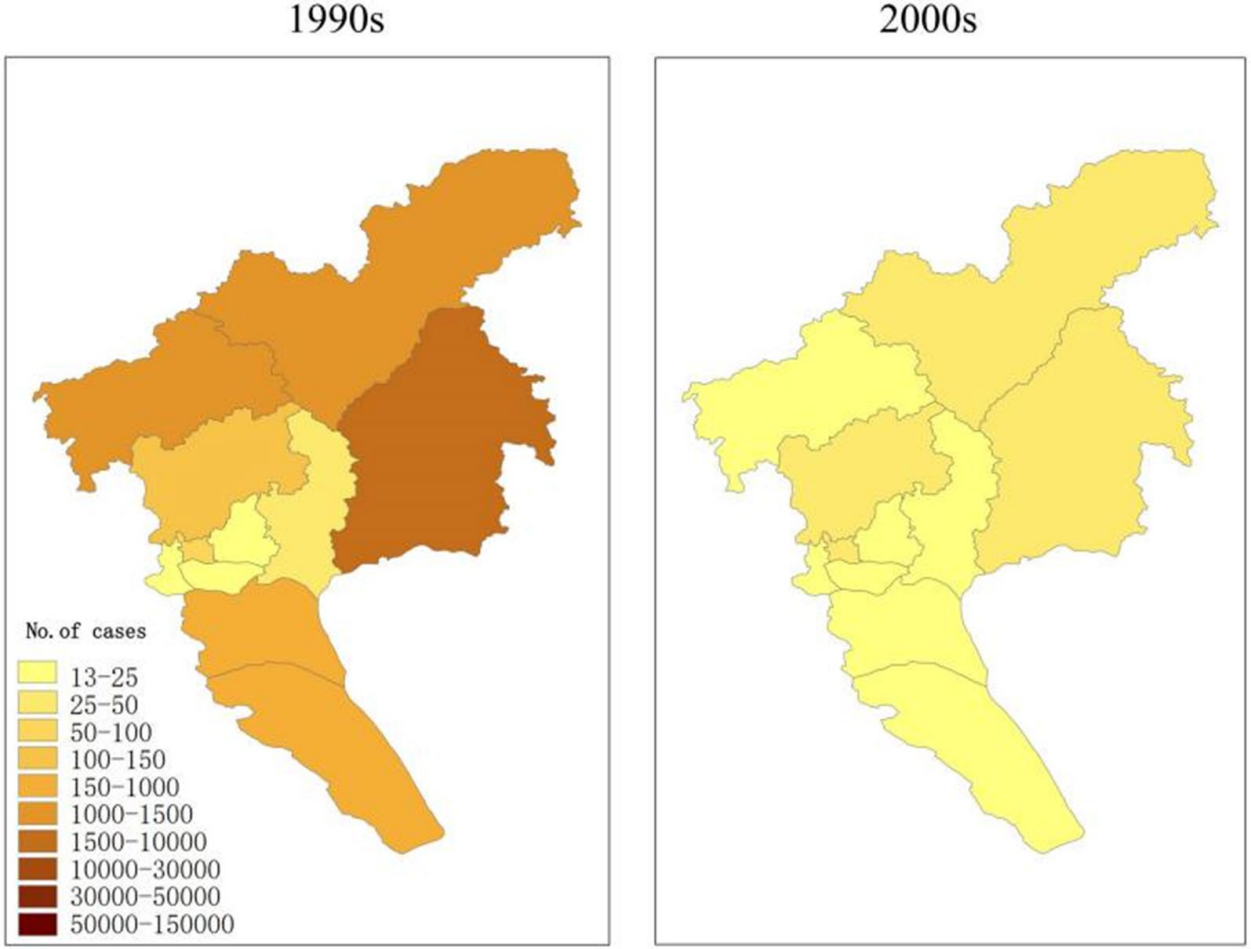
Geographical distribution of human malaria cases by decade in Guangzhou, China, 1950–2009

The geographic distribution of malaria cases in the Guangzhou region between 1950 and 2009. (Fig. 4). In the 1950s, cases mainly occurred in rural areas, with the highest number at 117,268 cases in the Zengcheng district, followed by 47,694 and 41,671 cases in the Conghua and Panyu districts, respectively. In the 1960s, the number of cases decreased significantly in all regions compared with the previous period, especially in the Conghua and Zengcheng districts; the highest number of cases was 23,364 in the Panyu district. In the 1970s, the number of cases in each region declined significantly. In the 1980s, the number of malaria cases increased compared with the 1960s owing to the increase in large-scale construction projects in Conghua, Huadu, and Zengcheng. During the early 1990s, a large number of migrant workers relocated to various districts (county-level cities) of Guangzhou, causing malaria outbreaks at some construction sites and mud and stone quarries where these workers were concentrated, with cases mainly occurring in the Conghua, Huadu, Zengcheng, and Baiyun districts (referred to as “three cities and one district”). This was the third malaria outbreak in Guangzhou since the founding of the People's Republic of China. The main malaria epidemic areas are in the mountainous towns along the “three cities and one district.” From 2000 to 2009, the incidence rate in each district decreased each year, and no outbreaks or local cases of falciparum malaria infection were reported in any district. Since the last case of local infection in the city was reported in 2008, no additional cases of local malaria infection have been reported in Guangzhou.

**Fig. 5:**
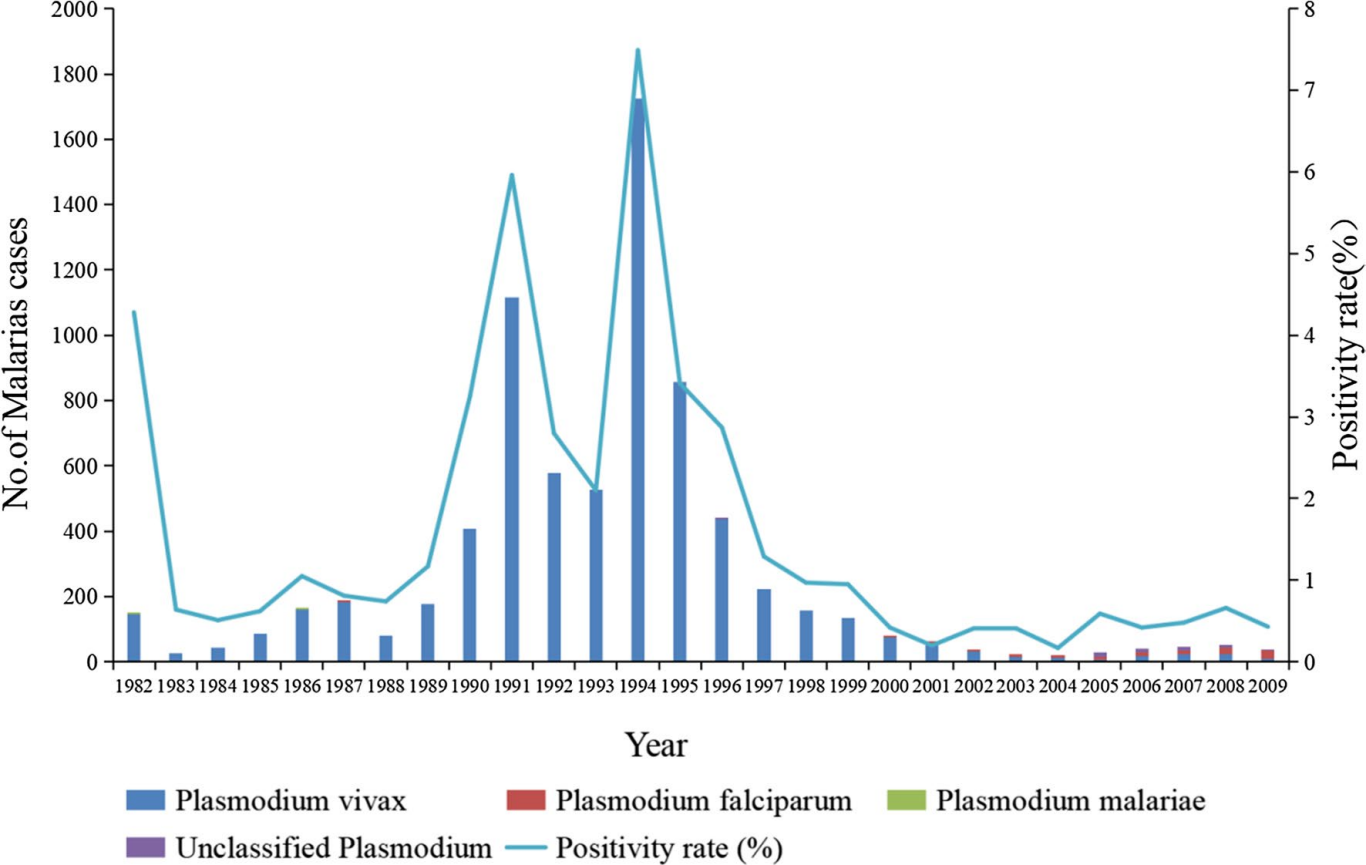
Positive blood test rates and *Plasmodium* classification for malaria in febrile patients in Guangzhou, China, 1982–2009

From 1982 to 2009, Guangzhou carried out testing for malaria parasites among patients with "four fevers" (clinical diagnosis of malaria; clinical suspicion of malaria; unexplained fever; a large number of patients with similar colds). The highest blood test positivity rate was 7.49% in 1994, followed by 5.96% in 1991 and 4.28% in 1983. Among positive patients from 1982 to 1989, the *Plasmodium* classification was dominated by *Plasmodium vivax* (99.67%), with small numbers of *P. falciparum* and *Plasmodium malariae*. Vivax malaria remained predominant in 1990–1999, with an increasing proportion of unclassified malaria. The number of positive cases has decreased significantly since 2000, with a predominance of *P. vivax* (67.33%) and *P. falciparum* (21.04%) malaria cases (Fig. 5). To determine the situation of *Anopheles* mosquitoes in endemic areas, several vector surveys were conducted in Guangzhou during 1982–2009, using the semi-overnight human baiting method to survey mosquito vectors at outbreak sites. A total of 7346 mosquitoes were caught during this period, with *Anopheles sinensis* accounting for the largest proportion (59.7%). *Anopheles minimus* and *Anopheles maculatus* have declined significantly since 1998; since then, there have been only a few years in which a small number of *An. minimus* and *An. maculatus* have been detected. In 1995, five monitoring points were selected in Guangzhou, with a small peak in the number of *Anopheles* mosquitoes caught.

**Fig. 6:**
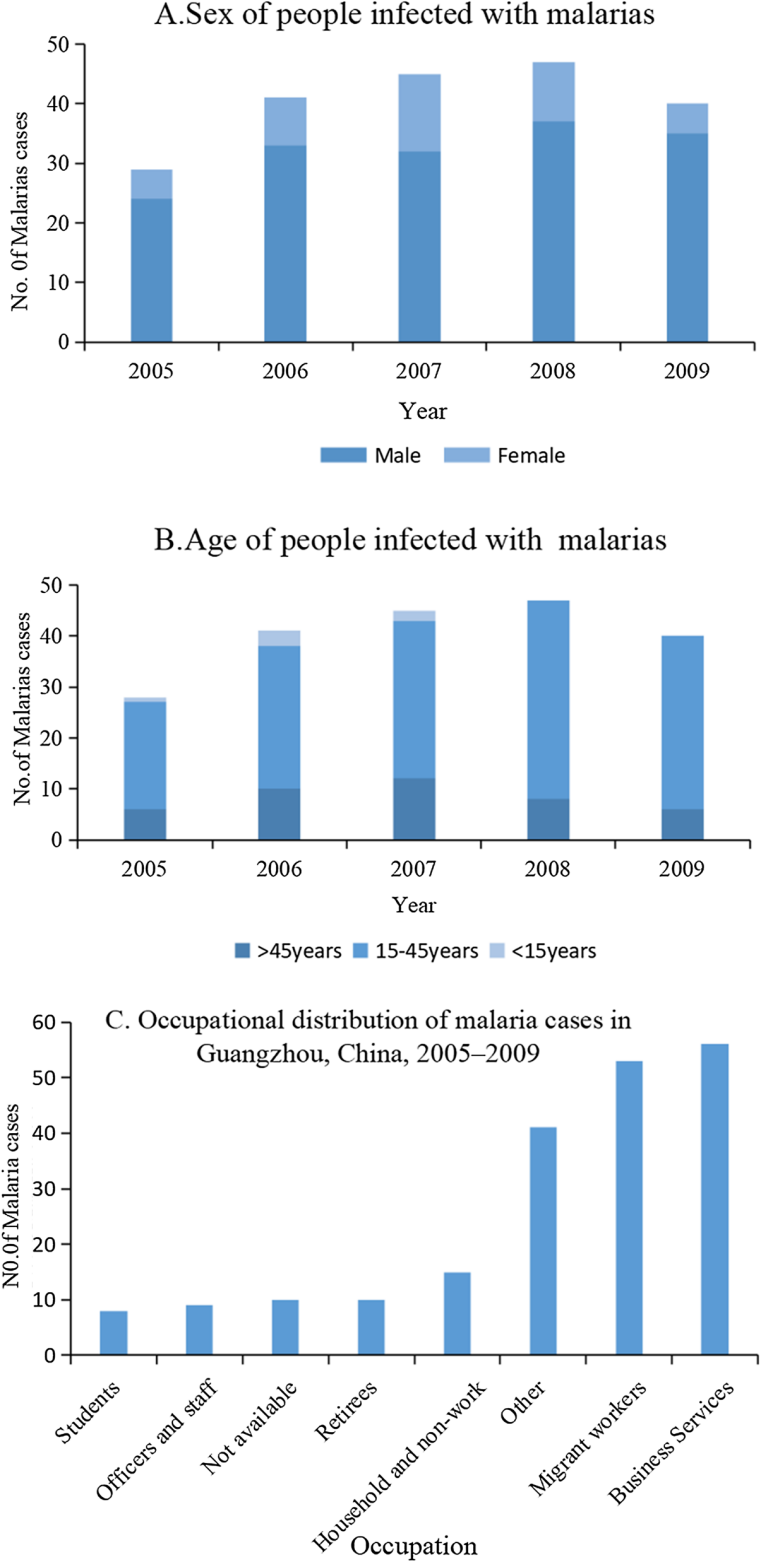
Demographic characteristics of malaria cases, Guangzhou, China, 2005–2009

According to the data on malaria cases collected by the Guangzhou Centre for Disease Control and Prevention, between 2005 and 2009, a total of 202 confirmed cases of malaria were reported, 161 (79.7%) in male and 41 (20.3%) in female individuals, with a sex ratio of 3.93:1 (Fig. 6A). The youngest reported case was 1 year old and the oldest was 83 years old, and cases were mainly concentrated in young adults. Among them, 153 cases (75.7%) were in the age group 15–45 years; 42 cases (20.8%) were in the age group > 45 years. The age group < 15 years had the fewest cases, with only 6 cases (Fig. 6B). The occupational profile shows that business services (27.72%) and migrant work (26.24%) are the groups with the highest incidence of malaria (Fig. 6C). Migrant work includes workers, farmers, and workers in urban areas with a rural residence (Fig. 6).

## Discussion

After more than 70 years of multi-sectoral cooperation efforts and the adoption of integrated prevention and control measures based on controlling the source of infection, Guangzhou has achieved improved results in the elimination of malaria. Guangzhou has had no local malaria infections since 2009, and in 2017, the city successfully passed the malaria elimination assessment and achieved the goal of malaria elimination [33, 34]. The malaria surveillance management system has been improved, surveillance of the *Anopheles* mosquito vector has been strengthened, the ability of medical staff to diagnose and treat malaria and malaria microscopy has been greatly improved, and public awareness of malaria control has been raised [25]. The establishment of this system involves the cooperation of an increasing number of organizations, which demonstrates the importance of collaborative communication among multiple sectors [35–37].

Interdisciplinary and joint multisectoral prevention and control are key measures to control malaria. In 2015, the European region was free of malaria. The number of countries moving toward malaria elimination continues to increase, with more countries submitting formal applications to the WHO for malaria-free certification [6]. A potential strategy for strengthening multisectoral collaboration is the implementation of malaria elimination within the United Nations system and establishment of a global, multisectoral health governance model [38, 39]. In these countries, malaria control is based on the following core strategies: (i) timely detection and reporting of cases and epidemiological investigations; (ii) prompt anti-malarial treatment after exposure; (iii) case and vector surveillance; and (iv) education and communication regarding malaria control.

Reducing the number and density of *Anopheles* mosquitoes is a key intervention for malaria control. *Anopheles* species and density surveys were conducted in the Baiyun and Conghua districts of Guangzhou in the 1960s, and window traps were used to collect *An. sinensis* specimens to analyse the mosquito’s habitat and behaviours. In 1965, Guangzhou City launched a public health campaign focusing on elimination of the “four pests” and the “three exterminations” (extermination of flies, rats, and hygienic dead angles). Sanitation teams were formed to dredge ditches and clean the city’s water network to reduce the breeding of flies. In 1971, Guangzhou City launched an autumn sanitation blitz, with aircraft spraying of large areas to eliminate mosquitoes and flies. Surveys of *Anopheles* densities and seasonal changes in malaria vectors on terraced surfaces in Huadu City and barren slopes in Baiyun District are crucial to the progress of malaria control.

Two other threats to malaria control in Guangzhou are the lack of knowledge about malaria and the management of malaria in mobile populations. For some time, people have not taken malaria seriously enough and have not paid sufficient attention to the threat of malaria, leading to the spread of the epidemic. A series of important decisions were introduced in the 1980s to improve knowledge among the general public in the fight against malaria. For example, mass education on malaria is carried out through the media, including wall banners and publicity in public squares. Strengthening community outreach efforts, enhancing health education for people entering and leaving the country, and providing health education courses on malaria prevention in primary and secondary schools are all important strategies that benefit high-risk groups, especially those in rural areas and mountain forests. Additionally, the increase in international business and trade exchange in Guangzhou in recent years has increased the risk of malaria transmission from and into the city, making it particularly important to improve the management of mobile populations. Establishing a sound information exchange mechanism, improving malaria protection for outbound personnel, strengthening malaria screening for inbound personnel, and providing malaria education for people on the move within the country are important elements of effective malaria prevention and control work.

There are several limitations in this study. During the 1950s, when the New China was first established, the case reporting and surveillance system was not well developed, and public knowledge about malaria was lacking, resulting in a large number of unreported cases and malaria deaths. There is a need to increase awareness about mosquito management and malaria prevention and control among the population.

After 72 years, Guangzhou now has a relatively well-established multi-sectoral joint prevention and control mechanism and post-elimination surveillance system. Efforts to control malaria have yielded substantial positive results with no local malaria infections in Guangzhou for many years. The present findings are intended to serve as a reference for other cities and countries that are experiencing malaria epidemics and to adapt and extend application to the control of other vector-borne diseases.

## Data Availability

Data will be made available on request.
